# A Peek into the Plasmidome of Global Sewage

**DOI:** 10.1128/mSystems.00283-21

**Published:** 2021-05-26

**Authors:** Philipp Kirstahler, Frederik Teudt, Saria Otani, Frank M. Aarestrup, Sünje Johanna Pamp

**Affiliations:** a Research Group for Genomic Epidemiology, Technical University of Denmark, Kgs. Lyngby, Denmark; Institute for Systems Biology

**Keywords:** plasmids, microbiome, wastewater, human, animals, Oxford Nanopore sequencing, plasmid reconstruction, Illumina NextSeq, Oxford Nanopore, plasmid DNA isolation

## Abstract

Plasmids can provide a selective advantage for microorganisms to survive and adapt to new environmental conditions. Plasmid-encoded traits, such as antimicrobial resistance (AMR) or virulence, impact the ecology and evolution of bacteria and can significantly influence the burden of infectious diseases. Insight about the identity and functions encoded on plasmids on the global scale are largely lacking. Here, we investigate the plasmidome of 24 samples (22 countries, 5 continents) from the global sewage surveillance project. We obtained 105-Gbp Oxford Nanopore and 167-Gbp Illumina NextSeq DNA sequences from plasmid DNA preparations and assembled 165,302 contigs (159,322 circular). Of these, 58,429 carried genes encoding for plasmid-related and 11,222 for virus/phage-related proteins. About 90% of the circular DNA elements did not have any similarity to known plasmids. Those that exhibited similarity had similarity to plasmids whose hosts were previously detected in these sewage samples (e.g., Acinetobacter, Escherichia, *Moraxella*, Enterobacter, *Bacteroides*, and Klebsiella). Some AMR classes were detected at a higher abundance in plasmidomes (e.g., macrolide-lincosamide-streptogramin B, macrolide, and quinolone) compared to the respective complex sewage samples. In addition to AMR genes, a range of functions were encoded on the candidate plasmids, including plasmid replication and maintenance, mobilization, and conjugation. In summary, we describe a laboratory and bioinformatics workflow for the recovery of plasmids and other potential extrachromosomal DNA elements from complex microbiomes. Moreover, the obtained data could provide further valuable insight into the ecology and evolution of microbiomes, knowledge about AMR transmission, and the discovery of novel functions.

**IMPORTANCE** This is, to the best of our knowledge, the first study to investigate plasmidomes at a global scale using long read sequencing from complex untreated domestic sewage. Previous metagenomic surveys have detected AMR genes in a variety of environments, including sewage. However, it is unknown whether the AMR genes were present on the microbial chromosome or located on extrachromosomal elements, such as plasmids. Using our approach, we recovered a large number of plasmids, of which most appear novel. We identified distinct AMR genes that were preferentially located on plasmids, potentially contributing to their transmissibility. Overall, plasmids are of great importance for the biology of microorganisms in their natural environments (free-living and host-associated), as well as for molecular biology and biotechnology. Plasmidome collections may therefore be valuable resources for the discovery of fundamental biological mechanisms and novel functions useful in a variety of contexts.

## INTRODUCTION

The term plasmid was introduced by Joshua Lederberg in 1952 to describe any extrachromosomal genetic particle (1). It was not until the 1970 that interest in plasmid research rapidly increased, and plasmids were introduced as cloning vectors into an area that was dominated by phages as a vector for the transfer of pieces of DNA of choice (2). Since then, plasmids have been highly valuable tools in molecular microbiology. In their natural environment, plasmids are considered key players in horizonal gene transfer. They play crucial roles in the ecology and evolution of bacteria, including their pathogenicity, since they can carry virulence factors such as toxins and antimicrobial resistance genes (3–6). However, the global diversity of plasmids and the distribution of antimicrobial resistance (AMR) genes have yet to be revealed.

The presence of AMR genes on plasmids is of major interest in the clinical and veterinary areas since they can render prescribed antibiotics for treating pathogens ineffective. There have been a range of large-scale metagenomic-based surveys of AMR genes in soils, humans, animals, plants, and sewage (7–12). However, the genomic context of AMR genes is largely unknown; for example, whether they are located in the bacterial genome or on plasmids. Such knowledge would be of great value to better assess their potential transmissibility rates and the global impact of AMR gene-carrying plasmids on human health.

Plasmids are usually circular DNA elements in bacterial cells, but they can also occur in linear form and be present in archaea and eukaryotic organisms. The size of plasmids is highly variable, ranging from 1,000 bases to hundreds of kilobases. They are present in different quantities (copy numbers) in bacterial cells, ranging from a single copy to hundreds of copies in a single cell. This intrinsic and unique nature of plasmids brings about several challenges in plasmidome research (i.e., research on the collective plasmid content in a sample). For example, a low plasmid/chromosome DNA ratio and potential low copy numbers can make it difficult to detect plasmids. These challenges are amplified when plasmidomes are examined from relatively low-cell-density environments such as wastewater. Even assembling and identifying plasmids with low copy numbers from high-biomass samples, including single isolates from whole-genome sequencing (WGS) data, can be challenging. To address these challenges, different approaches have been developed to increase the recovery of plasmids from WGS data (13–16).

Plasmids have now also been recovered from more complex microbiomes by using a number of strategies. These include multiple displacement amplification (MDA) with phi29 DNA polymerase prior to DNA sequencing (17), long-read sequencing technology of plasmid DNA, or the application of advanced assembly strategies (18–21). These studies, however, have been restricted to a single or only a few locations, and there is limited knowledge on the similarity and differences between plasmids from a range of geographical locations (22–26). We recently showed differences in the AMR gene profiles in urban sewage around the globe, but the locations of these AMR genes in the bacteria remain unknown (7).

To explore the plasmidome of global sewage, which is characterized by low bacterial cell numbers and challenges to isolate plasmid DNA, as previously shown (22–26), we employed here an optimized plasmid DNA isolation procedure, followed by both plasmid-safe DNase treatment and MDA to obtain sufficient plasmid DNA for Oxford Nanopore sequencing from global urban sewage samples. To improve plasmidome characterizations, we developed an assembly workflow, utilizing the long-read length from the Oxford Nanopore MinION sequencer and Illumina sequences. We obtained thousands of circular candidate plasmid sequences and explored their predicted functions.

## RESULTS

### Nanopore and Illumina sequencing output from plasmid DNA-enriched global sewage samples.

The sequencing of 24 plasmid-enriched DNA preparations from untreated sewage from five continents (Africa, Asia, Europe, North America, and South America) using Oxford Nanopore sequencing technology produced 1.2 to 9.7 Gbp (median, 3.5 Gbp) of sequencing data per sample (see Table S1 in the supplemental material). The median read length was 7.3 kb (range, 1,075 to 11,018 bases) (see Fig. S1 in the supplemental material). After quality trimming and removing sequences below 10,000 bases, the median sequencing throughput was 1.9 Gbp, and the median read length was 23,000 bases (see Table A at https://doi.org/10.6084/m9.figshare.13395446). The Illumina-generated sequencing data per sample were between 1.5 and 9.7 Gbp, with a median of 4.8 Gbp after adapter and quality trimming. A median of 41 million paired-end reads per sample were obtained (see Table B at https://doi.org/10.6084/m9.figshare.13395446).

10.1128/mSystems.00283-21.1 TABLE S1 Sewage sample information. Download Table S1, PDF file, 0.04 MB. Copyright © 2021 Kirstahler et al. 2021 Kirstahler et al. https://creativecommons.org/licenses/by/4.0/ This content is distributed under the terms of the Creative Commons Attribution 4.0 International license.

10.1128/mSystems.00283-21.2 FIG S1 Length of nanopore sequencing reads. The violin plot displays log**-**transformed read lengths. The horizontal dashed lines indicate log values for 1,000- and 10,000**-**base lengths, respectively. Most reads exhibit a read length below 10,000 bases, which is the cutoff value for our assembly workflow, and most of the reads are between 1,000 and 10,000 bases in length. Download FIG S1, TIF file, 0.6 MB. Copyright © 2021 Kirstahler et al. 2021 Kirstahler et al. https://creativecommons.org/licenses/by/4.0/ This content is distributed under the terms of the Creative Commons Attribution 4.0 International license.

### Circular DNA sequences obtained using single Oxford Nanopore reads.

**FIG 1:**
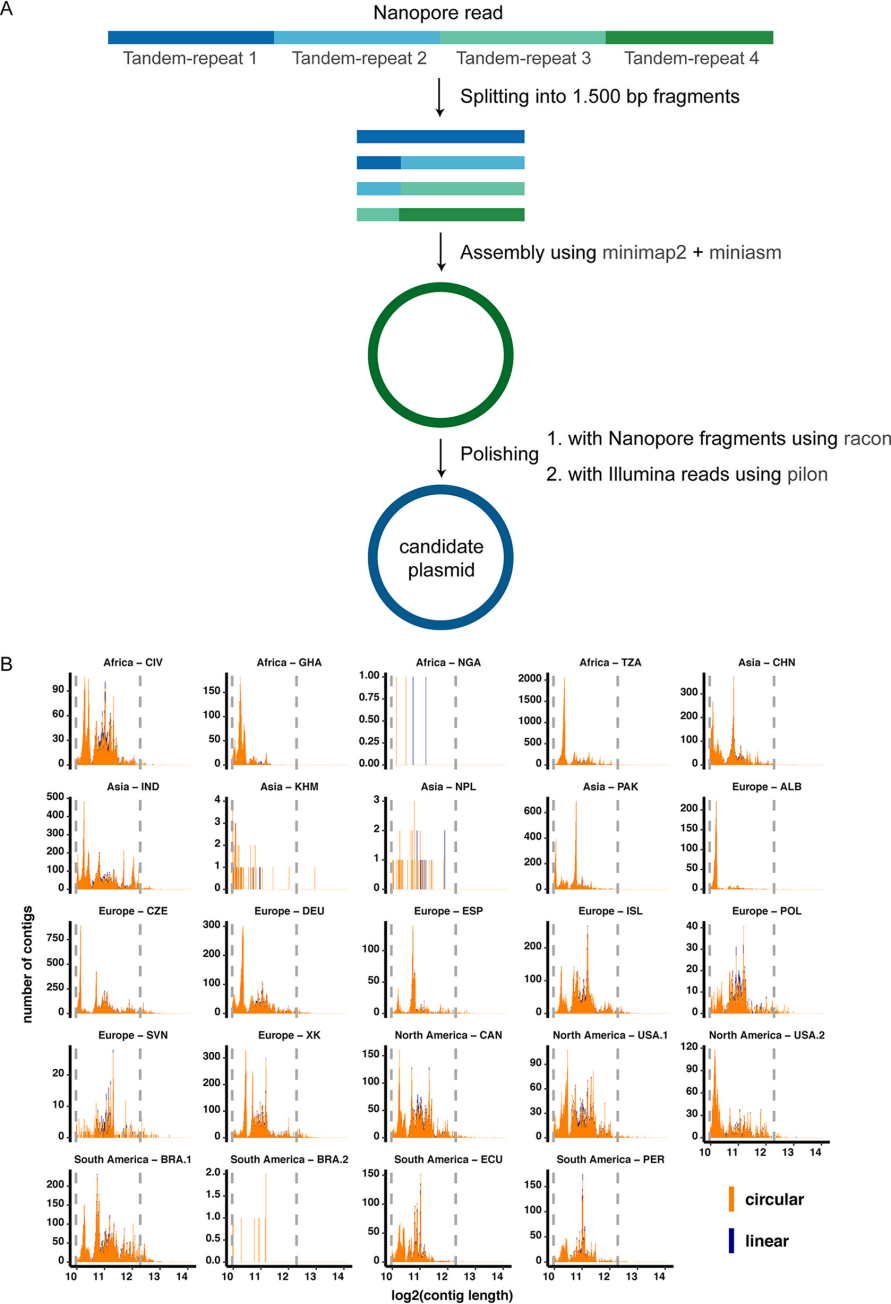
Schematic overview of the single read assembly workflow and size distribution of assembled reads. (A) Nanopore reads (based on plasmid DNA amplified with phi29) longer than 10,000 bases were split into 1,500**-**base fragments. The sequence fragments were then assembled using minimap2 and miniasm and subsequently polished two times: with the Nanopore fragments using racon and with the Illumina reads using pilon. (B) Size distribution of circular (orange) and linear (violet) assembled elements. These are the candidate plasmid sequences that successfully mapped to the original Nanopore read (i.e., covering more than 60% of the read and not overlapping by more than 50 bp for multiple hits). Of the total 165,302 assemblies, 159,322 were characterized to be circular and 5,980 were characterized to be linear.

Upon assembly and polishing (Fig. 1A), we obtained a total of 165,302 contigs from the 24 sewage samples, of which 159,322 contigs (96.4%) were suggested by using miniasm (27) to be circular (Fig. 1B; see also see Table C at https://doi.org/10.6084/m9.figshare.13395446). The longest assembled circular contig had a length of 17.4 kbp and was obtained from a sample in Brazil (BRA.1 [South America]). Most of the circular contigs were obtained from a Tanzanian (TZA [Africa]) sewage sample, and they had an average length of 1.7 kbp (see Table C at https://doi.org/10.6084/m9.figshare.13395446).

### Classification of assembled circular DNA elements.

**FIG 2:**
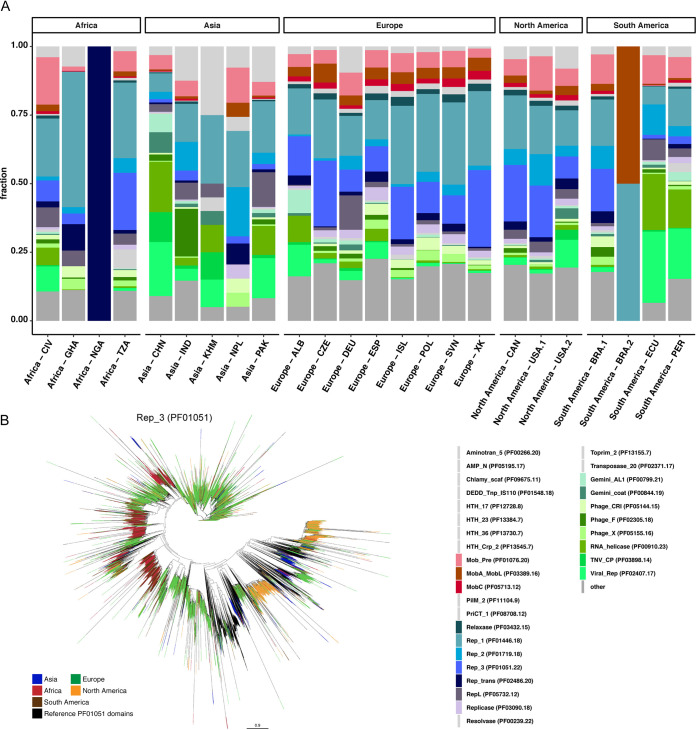
Functional characterization of circular DNA elements based on protein families. (A) Bar plot displaying the fraction of Pfam identifiers assigned to predicted proteins on the circular elements. The 31 Pfam identifiers represent the Top10 Pfam identifiers for each sample. Protein domains specifically involved in plasmid mobilization and plasmid replication are indicated in red and blue, respectively (see legend to the bottom right). Virus/phage-related Pfam identifiers are indicated in green. Remaining Pfam identifiers are grouped (other) and are indicated in dark gray. (B) Data for proteins with a Rep_3 (PF01051) domain (*n* = 24,824) were combined with the 1,637 reference Rep_3 (PF01051) proteins from Pfam. The protein sequences with a length of ≥40 amino acids (*n* = 16,930) were aligned using MAFFT. A phylogenetic tree was build using FastTree and visualized using FigTree. A high-resolution version of the phylogenetic tree is available from Figshare at https://doi.org/10.6084/m9.figshare.14112992.

To obtain information about the identity of the obtained circular DNA elements, we performed gene prediction, annotation, and classification based on plasmid- and virus/phage-specific Pfam domains (21). Overall, we detected Pfam domains (including domains of unknown function [DUF]) on 47.01% of the circular elements, potentially suggesting the presence of many novel DNA sequences not encoding known protein domains. For the DNA elements (circular and linear) for which Pfam domains were detected, the majority (88.39%) contained predicted genes with plasmid- or virus/phage-related Pfam entries (see Fig. 2; see also Fig. S2 in the supplemental material and Table D at https://doi.org/10.6084/m9.figshare.13395446). Overall, we found 55,337 circular DNA elements that encoded known plasmid-related Pfam domains (and not virus-related Pfam domains). The highest number of plasmid-related candidate sequences were detected in the sample from the Czech Republic (CZE [Europe]), followed by Tanzania (TZA [Africa]) and Kosovo (XK [Europe]). The sample from China (CHN [Asia]) was the only sample from which more potential virus/phage-related contigs than candidate plasmids were obtained (see Fig. 2; see also Fig. S2 in the supplemental material and Table D at https://doi.org/10.6084/m9.figshare.13395446).

10.1128/mSystems.00283-21.3 FIG S2 Plasmid- and virus (phage)-related circular DNA elements. The bar plots display the fraction (A) and total counts (B) of circular contigs containing Pfam IDs specific for plasmid and virus/phage-related proteins per sample. Each predicted protein by prodigal was searched against the Pfam databases using HMMER hmmscan and filtered for a *P* value of <0.00001. In a small subset of assemblies, we identify both viral and plasmid associated genes. Pfam IDs classified as “other than plasmid and viral” might still be plasmid relevant; they are just not specified as plasmid-related based on the stringent scheme used. Download FIG S2, TIF file, 0.5 MB. Copyright © 2021 Kirstahler et al. 2021 Kirstahler et al. https://creativecommons.org/licenses/by/4.0/ This content is distributed under the terms of the Creative Commons Attribution 4.0 International license.

On the circular elements with plasmid-related Pfam domains, protein families involved in plasmid replication were the most abundant, and these included Relaxase, Rep_1, Rep_2, Rep_3, Rep_trans, RepL, and Replicase (Fig. 2A). For example, we detected a total of 24,824 open reading frames with a plasmid replication initiator protein Rep_3 (PF01051) domain. Even though Rep_3 domain proteins from all continents were observed across the phylogenetic tree, some clades mainly represented proteins from one continent, interspersed with protein sequences from other continents (Fig. 2B). For instance, clades that mainly harbored proteins originating from Europe also frequently contained protein sequences from North America and other continents. Clades dominated by Rep_3 (PF01051) domain proteins from Africa also frequently harbored similar proteins from South America.

Furthermore, protein families involved in plasmid mobilization were also detected, such as Mob_Pre, MobA_MobL, and MobC (Fig. 2A). In addition, we identified protein families related to virus/phage replication and capsid proteins, as well as protein domains binding to DNA (HTH_17, HTH_23, and HTH_Crp_2), that might be involved in regulating gene expression.

### Global plasmidome pattern based on known plasmids.

**FIG 3:**
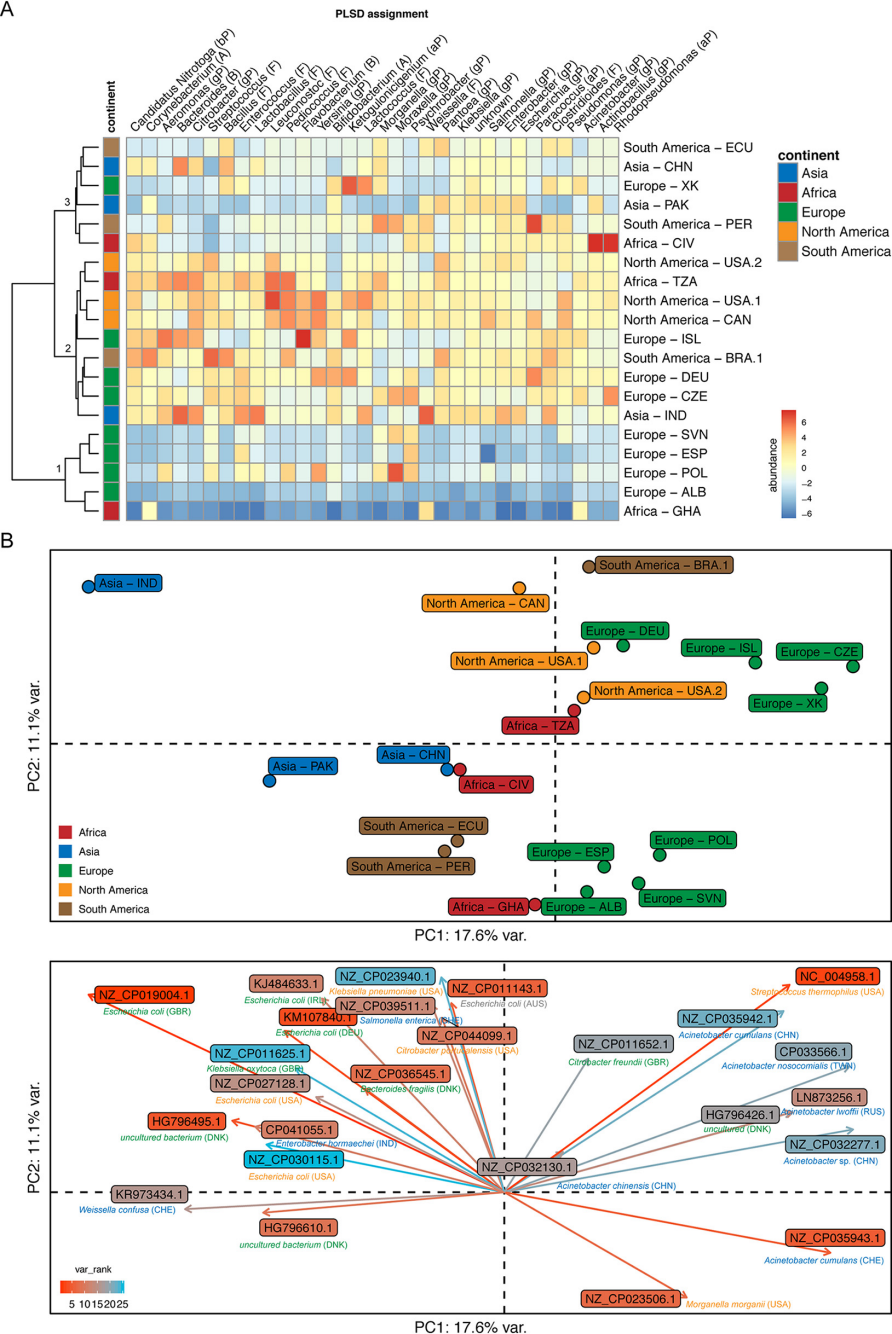
Comparison of candidate plasmids from global sewage with known plasmids in the plasmid database (PLSDB). (A) Heat map of centered log ratio (clr)-transformed abundancies of plasmid candidates assigned to plasmids in the PLSDB at the bacterial genus level. The phylum level is indicated by letters in in parentheses: A, *Actinobacteria*; B, *Bacteroidetes*; aP, *Alphaproteobacteria*; bP, *Betaproteobacteria*; gP, *Gammaproteobacteria*; F, *Firmicutes*. Clustering of samples was performed using the Euclidean distances of the clr-transformed values. (B) Principal-component analysis of clr-transformed abundancies of known plasmids detected by the PLSDB. The plot at the top reveals similarities and differences between samples. The plot at the bottom reveals the known plasmids that drive the partitioning of the samples, with 17.6% of the variation explained by the first and 11.1% by the second principal component.

To examine whether our collection of plasmid sequences contained already known sequences, we compared the obtained plasmid DNA sequences to the entries in the plasmid database (PLSDB). This analysis revealed that only 10.1% of our circular elements were similar to known plasmids (see Table E at https://doi.org/10.6084/m9.figshare.13395446). Most plasmids that exhibited some similarity to entries in the PLSDB originated from Acinetobacter (33%), *Enterococcus* (21%), and *Flavobacterium* (10%) spp., genera previously detected in these sewage microbiomes (7). Overall, most plasmids with similarities to already-known ones were found in samples from India, Kosovo, Pakistan, Czech Republic, Iceland, and Brazil (see Table E at https://doi.org/10.6084/m9.figshare.13395446). Clustering analysis of the abundancies of plasmids with known relatives in the PLSDB revealed three main clusters (Fig. 3A). The first cluster comprised samples that overall exhibited a low number of known plasmids and included samples from Europe (ALB, POL, ESP, and SVN) and a sample from Ghana. The second cluster included samples with plasmids from a large range of bacterial genera at higher abundance and comprised samples from Europe (ISL, DEU, and CZE), North America (USA.1, USA.2, and CAN), India, Brazil, and Tanzania. The third cluster comprised samples with known plasmids from few bacterial genera and included samples from Asia (CHN and PAK), Africa (CIV), Europe (XK), and South America (ECU and PER) (Fig. 3A).

In a principal-component analysis of the same data, a similar clustering was observed. Furthermore, along the first principal component, samples from Asia and Europe appeared to be most different from each other, with samples from Africa and from North and South America in between. Upon examining the particular reference plasmids and their bacterial hosts that were driving this pattern, a similar observation was made: plasmids from bacterial hosts originating from Europe appeared to segregate along the first principal component from plasmids and their bacterial hosts originating from Asia (Fig. 3B). This observation was supported by a cluster analysis on the plasmid level, in which five clusters were observed: samples from Europe did not cluster with samples from Asia, and different sets of known plasmids were found in the samples from Europe and Asia, respectively (see Fig. S3 in the supplemental material). Generally, only a few known plasmids were detected in the samples from Albania, Slovenia, Spain, Poland, Ecuador, and Ghana (see Fig. S3 in the supplemental material and Table E at https://doi.org/10.6084/m9.figshare.13395446).

10.1128/mSystems.00283-21.4 FIG S3 Comparison to known plasmids in plasmid database (PLSDB)—clustering on the individual plasmid level. Samples with fewer than 100 circular assembled contigs were removed from the analysis, as well as plasmids with fewer than 10 occurrences over all samples. Clustering of samples (columns) was done by using the Euclidean distances of the clr-transformed values. Download FIG S3, TIF file, 2.4 MB. Copyright © 2021 Kirstahler et al. 2021 Kirstahler et al. https://creativecommons.org/licenses/by/4.0/ This content is distributed under the terms of the Creative Commons Attribution 4.0 International license.

Given the large fraction of candidate plasmid sequences that did not exhibit similarity to already**-**known plasmids, we performed a reference-independent analysis by calculating MASH distances based on all plasmid sequences for each sample. In this analysis, the plasmidomes clustered in two main clusters (see Fig. S4 in the supplemental material). The first cluster harbored all samples from Europe (with the exception of Poland), as well as the samples from Canada (North America), Pakistan and India (Asia), and Côte d’Ivoire (Africa). The second cluster harbored all samples from South America and both samples from the United States (North America), as well as Tanzania and Ghana (Africa) and China (Asia) (see Fig. S4 in the supplemental material). This suggests that the sequence space encompassing novel plasmid sequences (i.e., those that did not exhibit similarity to sequences in the PLSDB) provides an extended, yet**-**to**-**be**-**discovered, perspective into plasmid ecology and evolution.

10.1128/mSystems.00283-21.5 FIG S4 Comparison between plasmidome samples—MASH distances. All plasmid candidate sequences for each sample from the five examined continents were sketched using MASH, distances were calculated, and results visualized using principal-component analysis. (A) Plot displaying the differences and similarities between all 24 plasmidome samples. (B) Plot displaying the differences and similarities between 22 plasmidome samples (all samples except NGA and BRA.2). Download FIG S4, TIF file, 0.8 MB. Copyright © 2021 Kirstahler et al. 2021 Kirstahler et al. https://creativecommons.org/licenses/by/4.0/ This content is distributed under the terms of the Creative Commons Attribution 4.0 International license.

### Antimicrobial resistance genes in plasmidomes.

To gain insight into antimicrobial resistance genes on the plasmids from sewage and compare them to those detected in the whole community of the same sewage samples, we performed a ResFinder analysis on three sequencing read data sets: whole-community DNA sequenced by using Illumina sequencing (7), plasmidome DNA sequenced by using Illumina sequencing (this study), and plasmidome DNA sequenced by using Nanopore sequencing (this study).

**FIG 4:**
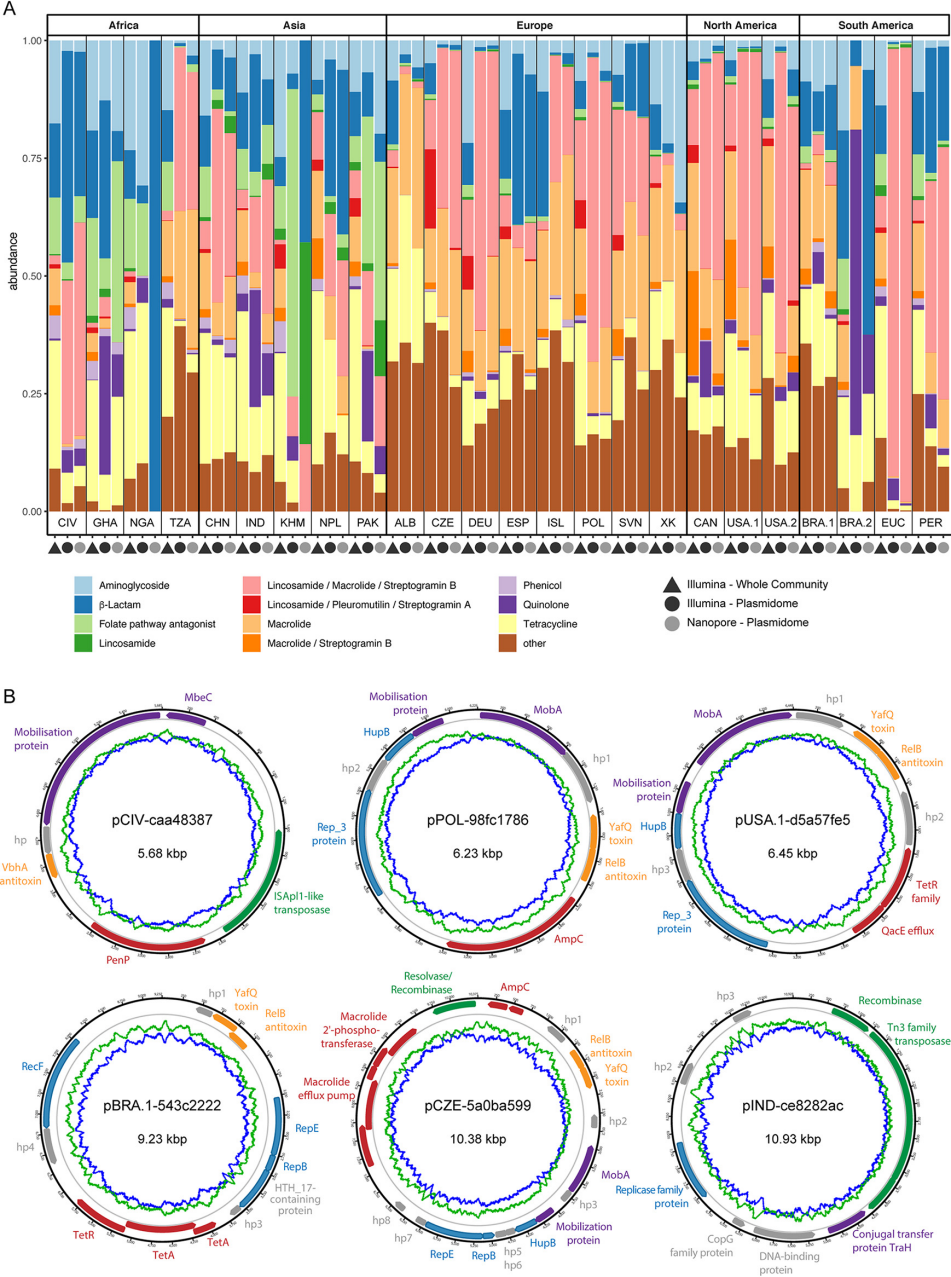
Antimicrobial resistance profiles from the whole community and plasmidomes from global sewage. (A) Bar plot displaying the proportions of antimicrobial resistance classes detected in a ResFinder-based analysis using the Illumina reads from the whole community, as well as Illumina reads from the plasmid preparations and Nanopore reads from the plasmid preparations. (B) Six examples of candidate plasmids are visualized in plasmid maps. The outermost black circle indicates the plasmid chromosome, and the coding sequence regions are colored according to their predicted function: replication (blue), mobilization (violet), transposition of DNA (green), antimicrobial resistance (red), toxin-antitoxin systems (orange), and hypothetical proteins (hp) and other proteins (gray). Blue and green lines indicate the GC and AT contents, respectively. The plasmids are named according to their origin: CIV (Côte d’Ivoire), POL (Poland), USA.1 (USA), BRA (Brazil), CZE (Czech Republic), and IND (India). Some sequencing errors might still be present in the candidate plasmid sequences, which are likely the reason why a few open reading frames are not properly predicted and appear fragmented, such as the gene encoding AmpC and macrolide efflux pump genes in the plasmid from Czechia. A detailed description about the plasmids is available from Figshare at https://doi.org/10.6084/m9.figshare.14039390.

Overall, many of the antimicrobial resistance genes and antimicrobial classes that were detected using whole-community sequencing were also detected in the two plasmidome data sets, with a few exceptions. For example, the two antimicrobial classes macrolide-streptogramin B and lincosamide-pleuromutilin-streptogramin A were not detected in the plasmidome samples in about half of the cases (Fig. 4A; see also Fig. S5A in the supplemental material and Tables F and G at https://doi.org/10.6084/m9.figshare.13395446). Occasionally, genes conferring resistance to other antimicrobial classes were also not detected in individual plasmidome samples compared to the whole community, and these included genes conferring resistance to lincosamide, phenicol, or aminoglycoside. It may be that genes that were detected more frequently in the whole-community sample, compared to the plasmidome samples, are preferentially located on bacterial chromosomes or larger plasmids.

10.1128/mSystems.00283-21.6 FIG S5 Heat maps depicting antimicrobial resistance profiles from the whole community and plasmidomes from global sewage based on presence/absence (A) and clr-transformed abundancies (B) of antimicrobial resistance gene classes. The antimicrobial resistance genes were identified in a ResFinder-based analysis using the Illumina reads from the whole community, the Illumina reads from the plasmid preparations, and the Nanopore reads from the plasmid preparations. Download FIG S5, TIF file, 2 MB. Copyright © 2021 Kirstahler et al. 2021 Kirstahler et al. https://creativecommons.org/licenses/by/4.0/ This content is distributed under the terms of the Creative Commons Attribution 4.0 International license.

Conversely, genes conferring resistance to the antimicrobial class macrolide-lincosamide-streptogramin B, as well as macrolides and quinolones, were more frequently observed in the plasmidome samples (Fig. 4A; see also Fig. S5 in the supplemental material and Tables F and G at https://doi.org/10.6084/m9.figshare.13395446). The most frequently observed AMR genes related to these three classes were *ermB*, *ermT*, and *ermF* (macrolide-lincosamide-streptogramin B); *mphE*, *mefA*, and *msrD* (macrolide); and *qnrB19*, *qnrD1*, *qnrD2*, *qnrD3*, and *qnrVC4* (quinolone). The higher frequency of these genes in plasmidome samples suggests that they are more frequently found on plasmids in general or on smaller plasmids compared to larger ones. Another gene frequently observed across samples, *msrE*, was slightly more abundant in plasmidomes (average abundance, 15.4%; standard error of the mean [SEM], 1.86%) compared to whole-community samples (average abundance, 11.5%; SEM, 1.88%). As examples, a few randomly chosen candidate plasmids and their corresponding genes, including AMR genes, are shown in Fig. 4B.

### Functional characterization of plasmidomes.

To gain further insight into the functions encoded on all circular elements, we obtained gene ontology (GO) annotations for the predicted proteins by mapping Pfam entries to GO terms. A clustering analysis revealed the separation of plasmidomes into two main clusters (see Fig. S6 in the supplemental material). Cluster 1 comprised samples from Europe (ISL, CZE, XK, and DEU), as well as North America (USA.1 and CAN) and South America (BRA.1 and ECU). Cluster 2 comprised samples from Asia (IND, PAK, and CHN), Africa (TZA and CIV), and the remaining samples from Europe (POL, ESP, and SVN) and South America (PER). This clustering based on protein functions appeared to have some similarity to the clustering based on nucleotide sequence similarity to known plasmids (Fig. 3). In both analyses, the European samples from ISL, CZE, and DEU exhibited similarities, while the other European samples from POL, ESP, and SVN clustered together separately. Furthermore, in both analyses samples from North America (USA.1 and CAN) and South America (BRA.1) clustered with the European samples (ISL, CZE, and DEU).

10.1128/mSystems.00283-21.7 FIG S6 Functional characterization of circular DNA elements—GO annotation. The heat map displays centered log ratio (clr)-transformed abundancies of GO annotations assigned to predicted proteins. Samples with fewer than 100 circular assembled contigs were removed from the analysis, as well as GO identifiers with fewer than 10 occurrences over all samples. The clustering of samples was performed by using Euclidean distances of clr-transformed values, resulting in two main clusters. Download FIG S6, TIF file, 2.1 MB. Copyright © 2021 Kirstahler et al. 2021 Kirstahler et al. https://creativecommons.org/licenses/by/4.0/ This content is distributed under the terms of the Creative Commons Attribution 4.0 International license.

Functions that appeared to be enriched in samples from cluster 1 include conjugation, recombinase activity, DNA methylation, protein secretion (type IV secretion system), response to antibiotic, toxic substance binding, response to toxic substance, unidirectional conjugation, and bacteriocin immunity (see Fig. S5 in the supplemental material). Cluster 2 appeared to have fewer proteins overall that could be annotated using this strategy, and the samples exhibited a higher diversity of functional patterns compared to samples from cluster 1. Some samples from cluster 2 exhibited an enrichment of proteins that may be related to viruses or phages, such as viral capsids, structural molecule activity, RNA binding, and RNA helicase activity, and these were, in particular, samples that that appeared to have a higher abundance of virus/phage-related Pfam domains (Fig. 2). Most samples in both clusters harbored proteins involved in plasmid maintenance (see Fig. S6 in the supplemental material).

## DISCUSSION

This is the first study to investigate plasmidomes on a global scale using long-read sequencing from sewage. We show that our approach facilitated the recovery of complete plasmids from complex metagenomic samples with sufficient quality to perform gene prediction and functional annotation. In total, we obtained 165,302 DNA elements, 159,322 of which were circular. The average length was 1.9 kb (minimum, 1 kbp; maximum, 17.4 kbp), suggesting that mainly small plasmids were obtained. This might reflect the true distribution but could also be biased due to a number of reasons; for example, smaller plasmids are more stable and thus have higher chance of getting through the DNA extraction step undamaged. Since a DNase step was used to reduce the amount of chromosomal DNA, damaged plasmids might have been digested as well. Another possibility could be that some plasmids were already damaged during storage and transportation, since the sewage was frozen and shipped, and many of the samples arrived thawed and were refrozen. Another reason could be that our assembly workflow was not able to perform a successful assembly on larger plasmids with a high number of tandem repeats.

We identified a range of functions encoded on the candidate plasmids, including plasmid replication and maintenance, mobilization, conjugation, antimicrobial resistance, and bacteriocin immunity. However, not all plasmid-related DNA elements encoded for a plasmid replication gene, suggesting that they may not be self-replicating DNA molecules. It should also be noted, however, that already-described plasmids do not necessarily bear a *rep* gene using current annotation algorithms. Furthermore, we found that about half of the circular DNA elements did not encode any known Pfam domains. This could suggest that we detected many novel DNA sequences not encoding known protein domains. One hypothesis is that a fraction of the circular DNA elements are novel extrachromosomal elements that have not yet been described and may also originate from various domains of life, including bacteria, archaea, and eukaryotes (28–30). Alternatively, open reading frames might not always have been properly detected because of sequencing errors not corrected in the polishing steps with the Nanopore and Illumina reads. This could certainly have contributed to this finding, since we occasionally observed fragmented genes due to remaining sequencing errors, even after polishing. This challenge may be alleviated by the ongoing improvement of Oxford Nanopore chemistry and base-calling algorithms. Nevertheless, we collectively obtained 58,429 DNA elements (circular and linear) that encoded proteins with plasmid-related Pfams, and 17,292 circular DNA elements exhibited sequence similarity to known plasmids, suggesting that we successfully discovered many novel candidate plasmid DNA sequences.

For candidate plasmids that exhibited some similarities to known plasmids, we found that they originated from bacterial taxa previously detected in complex sewage samples, such as Acinetobacter, Escherichia, *Moraxella*, Enterobacter, *Bacteroides*, and Klebsiella (7). These genera include bacteria that are part of the human gut microbiome and/or opportunistic pathogens. Hence, some of these plasmids might play a role in gut microbial ecology and potential AMR transmission (31, 32). It should be noted, however, that only ∼10.1% of our circular elements overall were similar to known plasmids in the PLSDB, and this may be partly explained by differences in plasmid contents (plasmid average sizes, 1.9 kbp [this study] and 53.2 kbp [PLSDB]) (33). In line with this, we observed that the plasmidome samples clustered somewhat differently when all candidate plasmid sequences were taken into account (and not only those that exhibited similarity to known reference plasmids). It will be interesting to investigate our candidate plasmids further in future studies, ideally through the involvement of more plasmidome samples and the use of extended metadata. There may be a range of factors that play role in explaining differences and similarities between plasmidomes, such as climate, population-related differences (including human ethnicity, health status, and sanitation), and economy, including trade between countries.

Overall, AMR classes that were detected in plasmidome sequencing data sets were also found in the sequencing data from the whole complex sewage samples, suggesting that the plasmidomes are a good representation of what is present in the complex samples. Some AMR gene classes, however, were more predominant in the whole community (e.g., macrolide-streptogramin B and lincosamide-pleuromutilin-streptogramin A), and others were more predominant in the plasmidomes (e.g., macrolide-lincosamide-streptogramin B, macrolide, and quinolone). This suggests that AMR genes conferring resistance to the latter AMR gene classes are preferentially located on plasmids compared to chromosomes. However, given that we mainly recovered small plasmids, this could also be an indication that the AMR genes preferentially detected in the whole community may be located on large plasmids that were not recovered here. Whether certain abundant AMR genes in the plasmidomes are plasmid or chromosome associated may also be dependent on the particular bacterial host (see Fig. S7 in the supplemental material) (34).

10.1128/mSystems.00283-21.8 FIG S7 Comparison of AMR genes with prevalence data by CARD (https://card.mcmaster.ca). The most frequently observed AMR genes that were more abundant in plasmidomes (compared to the whole-community sequencing data) were explored at the CARD website. Here, the prevalence for AMR genes is presented for a selection of pathogens, whether they are associated with the plasmid or chromosome. The prevalence data are calculated as follows: antimicrobial resistance (AMR) molecular prevalence data were generated using the resistance gene identifier (RGI), a tool for putative AMR gene detection from submitted sequence data using the AMR detection models available in CARD. To generate prevalence data, the RGI was used to analyze molecular sequence data available in NCBI genomes for 88 pathogens of interest. For each of these pathogens, complete chromosome sequences, complete plasmid sequences, and whole-genome shotgun (WGS) assemblies were analyzed individually by the RGI. The RGI results were then aggregated to calculate percent occurrence (see the report by Alcock et al. [34].) FIG S7, TIF file, 2.7 MB Copyright © 2021 Kirstahler et al. 2021 Kirstahler et al. https://creativecommons.org/licenses/by/4.0/ This content is distributed under the terms of the Creative Commons Attribution 4.0 International license.

While our approach and findings represent a significant advancement to previous work, there are still aspects that can be improved in the future. For example, the assembly workflow could be improved to resolve remaining repetitive regions within the plasmid, since a range of circular elements still consist of tandem repeats of the actual plasmid sequence. This could be resolved by introducing a dynamic cutting step using the *k*-mer composition of the full read. Despite the high error rate of the Nanopore sequencing reads, a raw read should still contain a set of *k*-mers 10 to 15 bases in length that could help to determine the appropriate fragmentation length. In addition, the plasmid DNA isolation could be improved significantly to increase (i) the overall amount of plasmid DNA (in order to avoid having to perform MDA) and (ii) the number of larger plasmids. Further possibilities to identify new plasmids could also involve *in vivo* proximity ligation Hi-C or single-cell sequencing that would also allow the discovery of new plasmids directly, together with their host cells (35, 36).

Overall, our study provides new insight into the technical applicability of long-read Nanopore sequencing for plasmidome analysis of complex biological samples, as well as a foundation for exploring plasmid ecology and evolution on a global scale. For example, we can now better explore the genomic context of AMR genes and show whether they are located on the microbial chromosome or on mobile genetic elements, such as plasmids. This knowledge is useful for assessing the potential transmissibility of AMR genes, thereby impacting antibiotic treatments in the medical and veterinary sectors and the One Health perspective. Furthermore, the data set provides a valuable resource for further exploring extrachromosomal DNA elements, including potential novel functions.

## MATERIALS AND METHODS

### Sample collection and preparation.

From the global sewage sample collection (7), we selected 24 samples from 22 countries (see Table S1 in the supplemental material). The samples originated from the five most populated continents on Earth and for which we had sufficient sample material available. From each sample, a sewage pellet was collected from 250 ml of untreated sewage by centrifugation at 10,000 × *g* for 10 min at 5°C. The sewage pellets were stored at −80°C until use.

### Plasmid DNA extraction and enrichment.

Plasmid DNA isolation was performed on individual sewage pellets (420 mg) by using a plasmid purification minikit (Qiagen, catalog no. 12123) with a Qiagen-tip 100 (Qiagen, catalog no. 10043) according to the manufacturer’s instructions with the following minor modifications: protein precipitation with P3 buffer mixture was incubated on ice for 15 min, elution buffer QF and EB buffer were preheated at 65°C prior to application, and the DNA pellet washing step was performed using ice-cold 70% ethanol after isopropanol precipitation. LyseBlue dye for cell lysis indication was added, and all buffer volumes were adjusted to the sewage pellet weight. The plasmid DNA pellet was dissolved in 35 μl of EB buffer for 1 h at room temperature. Linear chromosomal DNA was reduced by using Plasmid-Safe ATP-dependent DNase (Epicentre, USA) treatment for 24 h at 37°C according to the manufacturer’s instructions. The DNase was inactivated at 70°C for 30 min. To selectively enrich for circular DNA, the Plasmid-Safe DNase-treated DNA was amplified using phi29 DNA polymerase (New England Biolabs, USA) according to the manufacturer’s instructions, similar to as previously described (22). The plasmid DNA is amplified through rolling-circle amplification by the phi29 DNA polymerase using random primers, generating multiple DNA replication forks (17). This results in long DNA fragments that contain tandem copies (tandem repeats) of the same plasmid. Blank controls were used during plasmid DNA extractions and plasmid enrichment treatments. All negative controls had undetectable DNA measurements using Qubit double-stranded DNA (dsDNA) BR assay kit on a Qubit 2.0 fluorometer (Invitrogen, Carlsbad, CA).

### Plasmid DNA quality assessment.

The plasmid DNA yields from the sewage samples were evaluated by using gel electrophoresis and a Qubit dsDNA BR assay kit on a Qubit 2.0 fluorometer (Invitrogen). Plasmid DNA purity was measured and validated at absorbance ratios of 260/280 and 260/230 using a NanoDrop 100 (Thermo Fisher). During pilot experiments aimed at protocol development and plasmid DNA enrichment, we also assessed the quality of our plasmid DNA preparations using a 2100 Bioanalyzer (Agilent).

### Library preparation and Oxford Nanopore sequencing.

A 1-μg portion of plasmid DNA in 45 μl of buffer was used for library preparation. DNA was used without fragmentation. End repair and dA-tailing were performed using a NEBNext FFPE repair mix (New England BioLabs, catalog no. 6630) and an NEBNext Ultra II End Repair/dA-Tailing module (New England BioLabs, catalog no. 7546). DNA was mixed with 3.5 μl of NEBNext FFPE DNA repair buffer, 2 μl of NEBNext FFPE DNA repair mix, 3.5 μl of Ultra II End-Prep reaction buffer, and 3 μl of Ultra II End-Prep enzyme mix, and the volume was adjusted to 60 μl with nuclease-free water. The reaction tube was flicked three times, incubated at 20°C for 10 min, and then inactivated by heating at 65°C for 10 min. Clean-up was done using 60 μl of Agencourt AMPure XP beads. The bead reaction suspension was incubated on a HulaMixer at the lowest speed for 10 min, followed by two washes with freshly prepared 70% ethanol. DNA was then eluted from the beads in 61 μl of 65°C preheated nuclease-free water. A 1-μl DNA aliquot was assessed with a Qubit dsDNA BR assay to ensure that >700 ng was recovered. A volume of 60 μl of dA-tailed plasmid DNA was added to 25 μl of ligation buffer (LNB), 10 μl of NEBNext Quick T4 DNA ligase NEBNext Quick ligation module (New England BioLabs, catalog no. 6056), and 5 μl of adapter mix (AMX), and mixed by flicking the tube three to four times, followed by incubation at room temperature for an extended time of 1 h. The adaptor-ligated plasmid DNA was cleaned up by adding 40 μl of Agencourt AMPure XP beads, and the reaction was mixed by flicking the tube, followed by incubation on a HulaMixer at the lowest speed for 10 min. The beads were pelleted and resuspended twice in 250 μl of long fragment buffer LFB buffer (SQK-LSK109 kit; Oxford Nanopore Technologies). The cleaned adaptor-ligated DNA was eluted by incubating the pellet in 15 μl of elution buffer (SQK-LSK109 kit) for 20 min at room temperature, and then the supernatant was transferred to a new tube as a constructed library. Flow-cell priming and library loading preparation were performed according to the manufacturer’s instructions (SQK-LSK109 kit). Libraries were loaded on FLO-MIN106 R 9.4.1 Oxford Nanopore flow cells, and sequencing was performed for 48 h using MinKNOW software default settings.

### Illumina sequencing.

The enriched plasmid DNA samples were also subjected to Illumina NextSeq sequencing for downstream error correction of contigs. Libraries were prepared using a Nextera XT DNA library preparation kit (Illumina) according to the manufacturer’s instructions. The libraries were sequenced using a NextSeq 550 system (Illumina) with 2 × 150-bp paired-end sequencing per flow cell.

### Data processing.

Base-calling of Nanopore reads was performed using the guppy basecaller (v3.0.3+7e7b7d0) with the dna_r9.4.1_450bps_hac (high accuracy) configuration. Adapter trimming was performed using porechop (v0.2.3) downloaded from https://github.com/rrwick/Porechop using the default parameters. Illumina sequencing data were quality and adapter trimmed using bbduk from the bbmap suite (https://sourceforge.net/projects/bbmap/; v38.23) using the following settings: qin=auto, k=19, rref=adapters.txt, mink=11, qtrim=r, trimq=20, minlength=50, tbo, ziplevel=6, overwrite=t, and statscolumns=5.

### Plasmid assembly from single Nanopore reads.

Nanopore reads shorter than 10,000 bases were discarded. Each remaining read was cut into 1,500-base fragments and passed to the assembly step. The initial fragmentation step of the reads is needed since each read, amplified from a circular element during sample preparation, consists of multiple tandem repeats of the circular element. This is done to eliminate the tandem repeats and to increase the accuracy of the resulting candidate plasmid DNA sequence. We set the cutting threshold to 1.5 kb to balance between preserving the benefits of long-read sequencing and accounting for the error rate of Nanopore sequencing. We decided on a length threshold for cutting (i.e., 1.5 kbp) so as to not create candidate plasmid DNA sequences from small plasmids that contain multiple copies of the same plasmid. We set the cutting threshold to 1.5 kbp to balance between preserving the benefits of long-read sequencing and the error rate of Nanopore sequencing. We also preferred to keep the cutting threshold more toward the short range so as to not create candidate plasmids from small plasmids that contain multiple copies of the same plasmid sequence. Read fragments originating from one single read were assembled using minimap2 (v2.17-r943-dirty) in combination with miniasm version 0.3-r179 (parameter -s 800 bp) and error corrected using racon v1.3.3 (27, 37, 38). Assembled contigs were discarded if, after mapping the assembled contig back to the original Nanopore read, the hits did not span more than 60% of the read and if two hits overlapped by more than 50 bp. Assembled candidate contigs were error-corrected using five iterations of pilon v1.23 using the respective Illumina reads from the same sample (39). Candidate contigs longer than 1,000 bases were used for downstream analyses. A schematic overview of the method is presented in Fig. 1A.

### Global plasmidome analysis.

To examine the obtained plasmids from our global sewage collection in relation to already-known plasmids, we compared our obtained candidate plasmid DNA sequences to the DNA sequences in the plasmid database (PLSDB) using the webtool of PLSDB version 2019_10_07 (33). We used the search strategy “Mash screen” with a maximum *P* value of 0.005 and a minimum identity of 95%, as well as the optional “winner-takes-all” strategy. Samples with fewer than 100 circular assembled contigs were removed from the analysis, as well as genera with fewer than 10 occurrences over all samples. A clustering of samples was performed using the Euclidean distances of clr (centered log ratio)-transformed values.

Furthermore, all candidate plasmid sequences were sketched using MASH version 2.2 (40). The MASH distances between all samples were calculated using default settings, resulting in a 24-by-24 distance table that was used for principal-component analysis.

### Antimicrobial resistance gene detection analysis.

The trimmed Nanopore and Illumina reads were mapped against the ResFinder database (2020-01-25) using kma (version 1.3.0) (41, 42). Nanopore reads were mapped using the following settings: mem_mode, ef, nf, bcNano, and bc = 0.7. Illumina reads were mapped using the following settings: mem_mode, ef, nf, 1t1, cge, and *t* = 1. Resistance genes were counted across variants; for example, the alleles tet(A)_4_AJ517790 and tet(A)_6_AF534183 were both counted as *tet*(A). Centered log ratios were calculated using the pyCoDa package (https://bitbucket.org/genomicepidemiology/pycoda/src/master/).

### Gene prediction and functional analysis.

Gene prediction was performed using Prodigal version 2.6.3, and annotation of protein families was done by using hmmscan from HMMER3 version 3.3.1 (http://hmmer.org/) against the Pfam database version 33 (43, 44). Predicted genes, as well as functional annotation, were rejected if the *P* value was above 0.000001. Gene ontology (GO) annotations for Pfam IDs were acquired using the mapping of Pfam entries to GO terms, as described by Mitchell et al. (45).

To distinguish between potential plasmid and nonplasmid contigs, we used a scheme described previously (21). The scheme contains Pfam identifiers highly specific for plasmids and viruses. Proteins with a plasmid replication initiator protein Rep_3 (PF01051) domain (*n* = 24,824) were investigated further, together with the full set of reference Rep_3 domain proteins (*n* = 1,637) downloaded from Pfam (version 33.1). The two data sets were combined and Rep_3 domain proteins with a length of <40 amino acid residues were discarded, resulting in a data set of 16,930 Rep_3 (PF01051) domain proteins. The protein sequences were aligned using MAFFT (version 7.221) as part of the Galaxy platform (46, 47). A phylogenetic tree was then built using FastTree (version 2.1.10) (48) and visualized using FigTree (version 1.4.4) (https://github.com/rambaut/figtree/releases).

### Generation of plasmid maps.

The 50 longest assemblies from each sample were annotated using Prokka (49). Contigs of interest were chosen for mapping based on the presence of known plasmid-borne genes, such as replication and mobilization systems, toxin-antitoxin pairs, and AMR genes. Plasmids were inspected and visualized using DNAPlotter (50) and Geneious Prime version 2020.2.4. If a coding sequence from the Prokka analysis remained unannotated, it was manually annotated by using the BLAST search function against the nr database (51) and scanned with HMMER3 against the Pfam database, as described above.

### Data availability.

The DNA sequences generated in this project are available through ENA/GenBank/DDBJ under the accession number PRJEB41171 (Nanopore reads ERX4715074 to ERX4715097; Illumina reads ERX5299122 to ERX5299145; and assemblies ERZ1694234 to ERZ1694257). The code for the creation of assemblies is accessible from Github (https://github.com/philDTU/plasmidPublication), and additional supplemental material is available at https://figshare.com/projects/A_Peek_into_the_Plasmidome_of_Global_Sewage/94448.
