# Supplementary material for: A Peek into the Plasmidome of Global Sewage
Source: mSystems. 2021 May 26;6(3):e00283-21. doi: 10.1128/mSystems.00283-21 (PMC8269221; doi:10.1128/mSystems.00283-21)
Supplement: TABLE S1 [file msystems.00283-21-st001.pdf]

sample	continent	Country	city	extraction_date	plasmid_safe_dnase_date	phi20_date	sequencing_date	run_id
Africa - CIV	Africa	Côte d'Ivoire	Abidjan	25/03/2019	03/04/2019	04/04/2019	10/04/2019	GS_Plasmidome_13
Africa - GHA	Africa	Ghana	Tamale	25/03/2019	03/04/2019	04/04/2019	05/04/2019	GS_Plasmidome_4
Africa - NGA	Africa	Nigeria	NA	28/03/2019	15/04/2019	16/04/2019	06/05/2019	GS_Plasmidome_50
Africa - TZA	Africa	Tanzania	Moshi	01/04/2019	15/04/2019	16/04/2019	16/05/2019	GS_Plasmidome_15
Asia - CHN	Asia	China	Guangzhou	01/04/2019	15/04/2019	16/04/2019	13/05/2019	GS_Plasmidome_64
Asia - IND	Asia	India	Cochin	25/03/2019	03/04/2019	04/04/2019	05/04/2019	GS_Plasmidome_11
Asia - KHM	Asia	Cambodia	Phnom Penh	26/03/2019	03/04/2019	04/04/2019	12/04/2019	GS_Plasmidome_21
Asia - NPL	Asia	Nepal	Kathmandu	27/03/2019	15/04/2019	16/04/2019	16/05/2019	GS_Plasmidome_33
Asia - PAK	Asia	Pakistan	Karachi	25/03/2019	03/04/2019	04/04/2019	08/04/2019	GS_Plasmidome_7
Europe - ALB	Europe	Albania	Tirana	26/03/2019	03/04/2019	04/04/2019	10/04/2019	GS_Plasmidome_17
Europe - CZE	Europe	Czechia	Brno	27/03/2019	03/04/2019	04/04/2019	29/04/2019	GS_Plasmidome_23
Europe - DEU	Europe	Germany	Berlin	27/03/2019	03/04/2019	04/04/2019	29/04/2019	GS_Plasmidome_27
Europe - ESP	Europe	Spain	Barcelona	01/04/2019	15/04/2019	16/04/2019	20/05/2019	GS_Plasmidome_75
Europe - ISL	Europe	Iceland	Reykjavik	27/03/2019	03/04/2019	04/04/2019	01/05/2019	GS_Plasmidome_28
Europe - POL	Europe	Poland	Pu_awy	28/03/2019	15/04/2019	16/04/2019	03/05/2019	GS_Plasmidome_36
Europe - SVN	Europe	Slovenia	Ljubljana	28/03/2019	15/04/2019	16/04/2019	03/05/2019	GS_Plasmidome_38
Europe - XK	Europe	Kosovo	Prishtina	01/04/2019	15/04/2019	16/04/2019	08/05/2019	GS_Plasmidome_60
North America - CAN	North America	Canada	Toronto	26/03/2019	03/04/2019	04/04/2019	12/04/2019	GS_Plasmidome_22b
North America - USA.1	North America	United States of America	Portland,OR	01/04/2019	15/04/2019	16/04/2019	13/05/2019	GS_Plasmidome_74d
North America - USA.2	North America	United States of America	El Paso, Texas	01/04/2019	15/04/2019	16/04/2019	20/05/2019	GS_Plasmidome_74e
South America - BRA.1	South America	Brasil	Belo Horizonte	28/03/2019	15/04/2019	16/04/2019	08/05/2019	GS_Plasmidome_53
South America - BRA.2	South America	Brasil	Belém	28/03/2019	15/04/2019	16/04/2019	06/05/2019	GS_Plasmidome_53a
South America - ECU	South America	Ecuador	Galapagos	25/03/2019	03/04/2019	04/04/2019	08/04/2019	GS_Plasmidome_14a
South America - PER	South America	Peru	Lima	27/03/2019	15/04/2019	16/04/2019	01/05/2019	GS_Plasmidome_35
